# Efficacy and safety of vitamin E as adjunctive therapy for epilepsy: a systematic review and meta-analysis of randomized control trials

**DOI:** 10.3389/fneur.2025.1628032

**Published:** 2025-07-11

**Authors:** Yanfei Li, Gefei He, Juanjuan Huang, Lin Hu

**Affiliations:** ^1^Department of Pharmacy, The Affiliated Changsha Hospital of Xiangya School of Medicine, Central South University, Changsha, China; ^2^The First Hospital of Changsha, Changsha, China

**Keywords:** vitamin E, epilepsy, add-on therapy, seizure frequency, adverse events

## Abstract

**Objective:**

Vitamin E, functioning as an antioxidant, holds substantial potential in the adjuvant treatment of epilepsy. However, it remains uncertain whether the existing evidence is adequate to validate the use of vitamin E as an add-on therapy for improving epilepsy outcomes. The aim of this study was to explore the efficacy and safety of vitamin E as an adjuvant treatment for epilepsy.

**Methods:**

We searched PubMed, Embase, the Cochrane Library, and Chinese databases including the Chinese Biomedicine Literature Database, China National Knowledge Infrastructure, Chinese Sci-tech Journal Database, Wanfang Data for eligible studies from inception to February 28, 2025. Meta-analysis was performed to calculate the risk ratio (RR) and weighted mean difference (WMD) of the included randomized controlled trials (RCTs).

**Results:**

Among the 2,348 records obtained, 11 RCTs involving 824 patients were included after literature screening. Vitamin E had a potential advantage in reducing seizure frequency by >75% (RR = 1.73, 95% confidence interval (CI) (1.31, 2.28), *p* < 0.01), compared with the control group. Subgroup analysis showed a statistically significant difference in the reduction of seizure frequency by >50% (RR = 1.58, 95% CI (1.27, 1.96), *p* < 0.01) between the vitamin E group and the control group, especially in children (RR = 1.69, 95% CI (1.29, 2.20), *p* < 0.01). The plasma total antioxidant capacity was higher (WMD = 3.03, 95% CI (2.65, 3.40), *p* < 0.01) while the malondialdehyde levels were lower (WMD = −6.28, 95% CI (−8.01, −4.54), *p* < 0.01) in the vitamin E group than in the control group. There was no statistically significant difference in the rates of total adverse events (RR = 0.97, 95% CI (0.93, 1.02), *p* = 0.25).

**Conclusion:**

Vitamin E shows potential as adjunctive therapy, particularly in pediatric populations, with acceptable safety profile, but high-quality trials are required to confirm its efficacy and safety.

## Introduction

Epilepsy is a chronic neurological disorder characterized by recurrent seizures. According to the Global Burden of Disease Study in 2021, the number of people with active epilepsy worldwide has reached 51.7 million, with an age-standardized prevalence of 658 per 100,000. More than 80% of these patients are concentrated in low-income and middle-income countries (1). Anti-seizure medications (ASMs) are the most important and fundamental treatment. Although traditional antiepileptic drugs such as carbamazepine and valproic acid are still the first-line clinical medications, their limitations are significant. Firstly, more than 30% of patients have been shown to be resistant to antiepileptic drugs, which is associated with severe morbidity and increased mortality (2, 3). Secondly, long-term use of ASMs may be accompanied by general adverse reactions such as drowsiness, gastrointestinal discomfort, changes in personality and behavior, as well as some potentially serious adverse reactions such as myelosuppression, Stevens-Johnson syndrome and teratogenicity (4). Therefore, it is particularly important to explore safe and effective new adjuvant treatment strategies.

Recent research highlights oxidative stress, mitochondrial dysfunction, and neuroinflammation as key factors in epilepsy’s pathophysiology (5). During seizures, elevated brain oxidative stress triggers abnormal neuronal discharges and cell death (6). Vitamin E, a fat-soluble antioxidant, exerts neuroprotection via scavenging free radicals, regulating the Nuclear factor erythroid 2 - related factor 2 (Nrf2) pathway, suppressing pro- inflammatory factor release, and stabilizing mitochondrial membrane potential (7–10). In animal models, it mitigates seizures and brain injury by curbing free radical production (11, 12). Clinical findings on vitamin E’s efficacy in epilepsy are inconsistent. Ogunmakan et al.’s randomized double-blind trial showed over 60% of pediatric epilepsy patients had reduced seizure frequency after 3-month vitamin E-antiepileptic drug combination therapy (13). Mehvari et al. (14) reported decreased seizure frequency and electroencephalogram (EEG) improvement in adult patients after 6-month vitamin E supplementation. Conversely, Raju et al.’s crossover trial found no significant impact on seizure frequency (15). Additionally, individual variability may affect vitamin E’s efficacy, and in some cases, it may not notably improve seizures or the condition (16).

Currently, no systematic review has comprehensively synthesized the clinical evidence of vitamin E as an adjuvant for epilepsy. This study systematically searched Chinese and English databases and used meta-analysis to quantitatively assess vitamin E’s effects on seizure control, antioxidant capacity and safety, aiming to offer an evidence-based foundation for clinical decision-making.

## Materials and methods

### Search strategy

We conducted a systematic search of multiple authoritative databases. The English databases included PubMed, Embase, and the Cochrane Library, whereas the Chinese databases consisted of the Chinese Biomedical Literature Database (CBM), the China National Knowledge Infrastructure (CNKI), Wanfang Data, and the Chinese Sci-Tech Journal Database (VIP database) for randomized controlled trials (RCTs). The search period extended from the inception of each database to February 28, 2025. Moreover, we manually scoured the reference lists of included studies to unearth relevant trials. The search utilized the keywords “vitamin E or alpha-tocopherol” and “epilepsy or seizure.” Articles were restricted to those written in English and Chinese. Data from gray literature was excluded from the search because we had no access to it. A comprehensive search strategy can be located in Supplementary Table S1.

### Inclusion and exclusion criteria

Studies were eligible for inclusion in this systematic review if they met the following criteria: (i) Patient population: all enrolled patients had a diagnosis of epilepsy. (ii) Intervention and control: the studies compared the efficacy and safety of vitamin E against a blank or placebo control. No restrictions were imposed on the dosage and duration of vitamin E administration. (iii) Study design: only randomized controlled trials (RCTs) were considered. (iv) Outcome measures: seizure frequency reduction was the primary outcome. Secondary outcomes covered plasma total antioxidant capacity (T-Aoc) and malondialdehyde (MDA) levels, EEG alterations, and the occurrence of adverse events.

Studies were excluded when outcome measurements were ambiguous, trial designs were non-randomized, research involved combined drugs rather than vitamin E alone, full-text data was unavailable, or research did not revolve around vitamin E.

### Literature screening and data extraction

Two researchers independently screened literature and extracted data following pre-defined criteria and search strategy. Using NoteExpress software, we removed duplicates, reviewed titles and abstracts to exclude ineligible studies, read full-texts, determined eligibility, and created an extraction table. Data extracted covered the first author, publication year, sample-related details, acceptance standard, intervention, comparator, treatment duration and outcome measures. When ambiguity or disagreement occurred in literature selection or data retrieval, we resolved issues through joint discussions or consultations with an additional researcher. Our study adhered to the Preferred Reporting Items for Systematic Reviews and Meta-Analyses (PRISMA) guidelines (17), and all relevant items on the PRISMA checklist were addressed. Given that this systematic review and meta-analysis solely involved pre-published, anonymized data, ethical approval was waived.

### Deviation risk assessment

Risk of bias assessment for the included studies was carried out by two evaluators. The assessment adhered to the guidelines set out in the Cochrane risk of bias assessment tool (18). Multiple key domains were evaluated, including random sequence generation, allocation concealment, blinding procedures, integrity of outcome data, selective reporting, and other potential sources of bias. Based on the pre-defined scoring criteria, each study was classified as having a low, unclear, or high risk of bias. In cases where discrepancies arose during the evaluation, a third evaluator was consulted. The final risk level of each study was determined following this consultative process.

### Quality assessment

Two evaluators independently utilized the GRADEpro Guideline Development Tool framework for a systematic assessment of the evidence quality. They also collaborated with a separate third party to tackle and resolve any complications present in the evaluation process. The assessment was based on various factors, namely the risk of bias, inconsistency, indirectness, imprecision, and other considerations. After the assessment, the quality of the evidence was rated as high, moderate, low, or very low.

### Statistical analysis

A meta-analysis was carried out by two researchers through the statistical analysis of data from two or more RCTs with congruent outcome measures, with the assistance of Review Manager 5.3 software. For dichotomous variables, the pooled effect size was measured as the risk ratio (RR) accompanied by 95% confidence intervals (CIs). In the case of continuous data, the weighted mean difference (WMD) with 95% CIs served as the metric for effect size. *p* < 0.05 was considered statistically significant. To evaluate heterogeneity among studies, the Mantel–Haenszel chi-square test, set at an *α* level of 0.1, and the *I^2^* statistic were deployed. When the *p* value ≥ 0.1 or *I^2^* < 50%, it signified low heterogeneity, justifying the application of a fixed-effect model in meta-analysis. Conversely, a *p* value < 0.1 and an *I^2^* ≥ 50% indicated high heterogeneity. Under such circumstances, a random-effects model for meta-analysis, subgroup analysis, or sensitivity analysis was considered appropriate. When the meta-analysis of outcomes involved fewer than 10 studies, we evaluated publication bias using the fail-safe number (Nfs). The Nfs was calculated following Rosenthal’ s formula: Nfs_0.05_ = (∑Z/1.645)^2^-k, where k represents the number of studies included in the meta-analysis, and Z corresponds to the *Z* value of each individual study. For each specific outcome indicator, the data were summarized, followed by the implementation of a descriptive analysis.

## Results

### Literature screening

**Figure 1:**
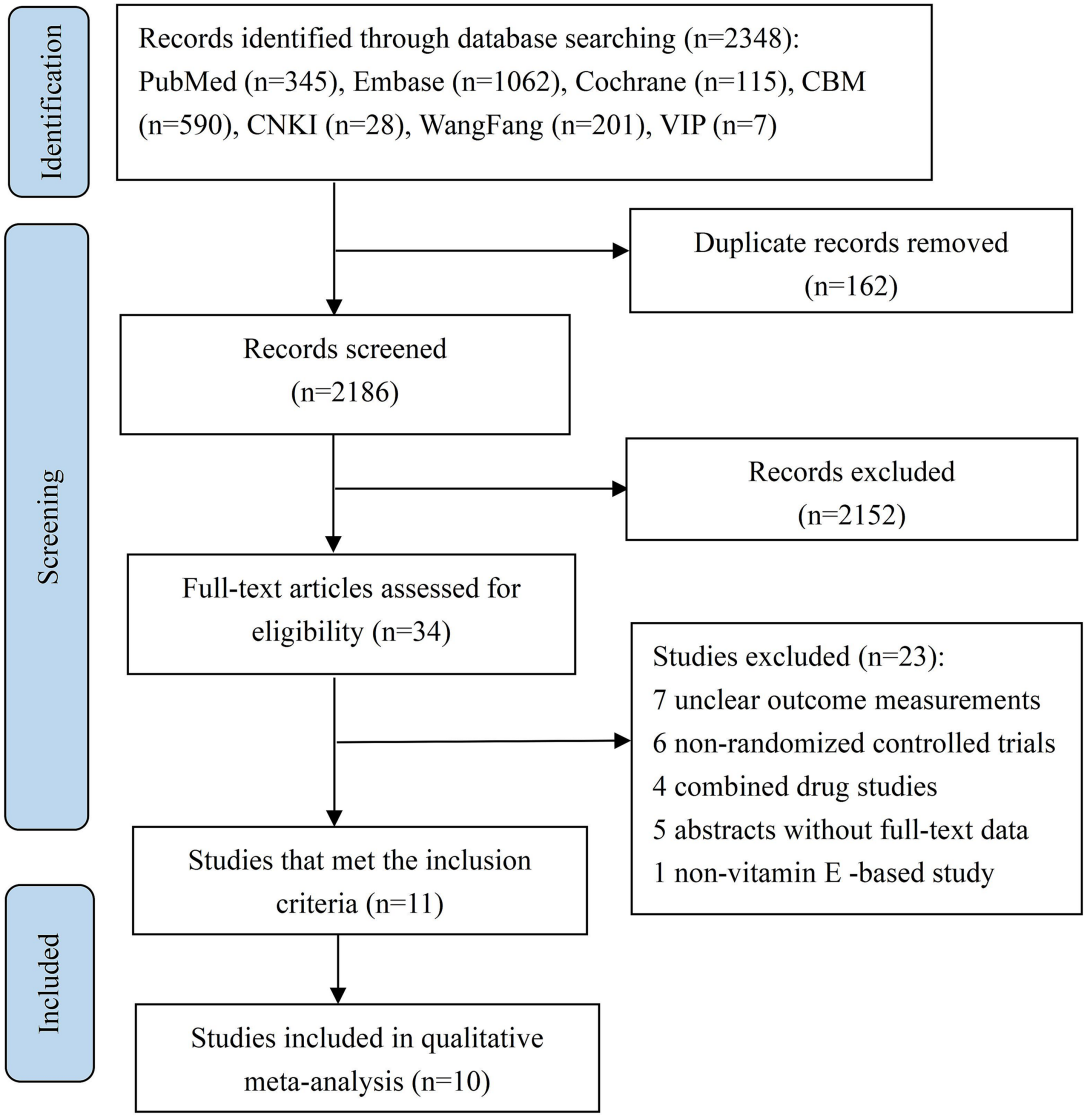
Literature retrieval and screening procedure.

This study began with the retrieval of 2,348 articles. Using NoteExpress software, we eliminated 162 duplicates. The remaining pool of articles underwent a title and abstract screening, during which 2,152 articles were excluded. Next, full-text assessment of the 34 remaining articles was carried out. Based on pre-defined inclusion and exclusion criteria, 23 articles were removed from consideration. Ultimately, 11 articles met all requirements and were incorporated into the study. Among these, 10 articles were selected for qualitative synthesis meta-analysis. Figure 1 provides a comprehensive overview of the search and screening methodology employed in this study.

### The characteristics of included studies

Of the 11 included RCTs, four were published in English (13–15, 19) and seven in Chinese (20–26). The treatment duration of vitamin E ranged from 4 weeks to 24 weeks. Six RCTs reported the proportion of female patients, which ranged from 39.4 to 47% (13, 14, 21–24). Four RCTs reported the number of dropouts (2 (13), 1 (14), 5 (15) and 4 (19) patients respectively) and no participants dropped out in the other 7 (20–26) Chinese RCTs. Eight studies (13–15, 19–21, 23, 26) included drug-refractory patients, while the other three studies (22, 24, 25) did not specify patients’ drug responsiveness. Four studies (13, 15, 20, 22) specified the seizure types of included patients. The characteristics of the included RCTs are shown in Table 1.

**Table 1:** Characteristics of included studies.

Study	Inclusion criteria	Sample size (VE/C)	Male/female		Age/years old		Intervention		Duration/weeks	Outcome measures
			VE	C	VE	C	VE	C		
Gao et al. (20)	1^bc^, 2^a^, 3^a^, 4^e^	37/40	NR	NR	5 ~ 14	5 ~ 14	400 mg qn + AEDs	AEDs	12	①③⑨
Mehvari et al. (14)	3^b^, 4^ef^, 6	32/33	19/13	19/14	28.8 ± 5.3	28.6 ± 8.8	400 IU(294 mg) qd + AEDs	placebo+AED	24	②③④⑤⑥⑦
Ogunmekan and Hwang(13)	2^ad^, 3^d^, 5^b^	12/12	7/5	6/6	6 ~ 17	6 ~ 17	400 IU(294 mg) qd + AEDs	placebo+AEDs	12	①③
Raju et al. (15)	1^d^, 2^ac^, 3^c^, 4^c^	43^*^/43^*^	NR	NR	>12	>12	600 IU(441 mg) qd + AEDs	placebo+AEDs	24	①⑨
Sullivan et al. (19)	3^d^, 4^de^	18/17	NR	NR	NR	NR	250 IU(184 mg) qd + AEDs	placebo+AEDs	24	①⑧
Wang et al. (21)	1^abce^, 3^a^, 4^ab^, 5^a^, 6	67/67	39/28	41/26	18 ~ 65	18 ~ 65	100 mg qd + LEV	LEV	12	①③④⑤⑨
Wang (22)	1^abcd^, 2^b^	33/33	20/13	18/15	0.67 ~ 14	0.67 ~ 14	≤2y:100 mg qod + AEDs >2y:100 mg qd + AEDs	AEDs	12	①④⑤⑨
Wang (23)	1^bc^, 3^b^, 4^a^, 6	53/53	33/20	31/22	18 ~ 59	18 ~ 57	100 mg qd + LEV	LEV	4	①③④⑤⑨
Zhang et al. (24)	1^abcd^	50/50	25/25	28/22	1 ~ 12	1 ~ 12	≤2y:100 mg qod + LTG >2y:100 mg qd + LTG	LTG	12	①②③
Zhang (25)	1^abcd^	45/45	NR	NR	0.75 ~ 11	0.75 ~ 11	≤2y:100 mg qod + AEDs >2y:100 mg qd + AEDs	AEDs	12	①③④
Zhong et al. (26)	3^d^, 4^e^	40/40	NR	NR	4.9 ~ 12.7	4.9 ~ 12.7	10 mg/kg/d qd + AEDs	AEDs	24^#^	①⑨

Age presented as mean ± SD or range. VE, the vitamin E group. C, the control group. AEDs, antiepileptic drugs. LEV, levetiracetam. LTG, Lamotrigine. *NR, not report.* Y, years old. ① reduction of seizure frequency, ② seizure frequency, ③ electroencephalogram (EEG), ④ Total antioxidant capacity (T-Aoc), ⑤ malondialdehyde (MDA), ⑥ catalase, ⑦ glutathione, ⑧ Serum vitamin E level, ⑨ adverse events. *, patients were crossed over to the second treatment phase after a 1-week washout period. ^#^Follow-up for more than half a year.

1. Diagnosis: ^a^, all of patients met the diagnostic criteria for epilepsy and its seizures formulated by the International League Against Epilepsy (ILAE). ^b,^ all patients have obvious clinical manifestations of epilepsy. ^c^, all patients have obvious EEG characteristics of epilepsy. ^d^, absence of a progressive/regressive. brain lesion/other neurological diseases as evaluated by head computed tomography (CT) scan or magnetic resonance imaging (MRI). ^e^, the course of disease is greater than or equal to 3 months.

2. Seizure types: ^a^, generalized tonic–clonic seizures. ^b^, partial seizures. ^c^, simple partial, complex or secondarily generalized seizures. ^d^, other stable seizure pattern that were clearly distinguished.

3. Seizure frequency: ^a^, more than one a month. ^b^, more than two a month. ^c^, more than three a month in the preceding 3 months. ^d^, more than four a month.

4. Antiepileptic drug regimens: ^a^, the AEDs are not effective. ^b^, stable AEDs regimen. ^c^, adequate and appropriate therapy for at least 6 months with not more than two AEDs. ^d^, exhibit drug side effects related to polytherapy. ^e^, taking various AEDs for at least half a year. ^f^, no vitamin E supplementation for 6 months prior to study entry.

5. Compliance: ^a^, subjects can strictly follow the doctors’ advice to take medicine and accept regular follow-up. ^b^, subjects had attended the clinic for at least 6 months.

6. All patients voluntarily signed the informed consents, and the study was approved by the ethics committee.

### Risk of bias assessment

**Figure 2:**
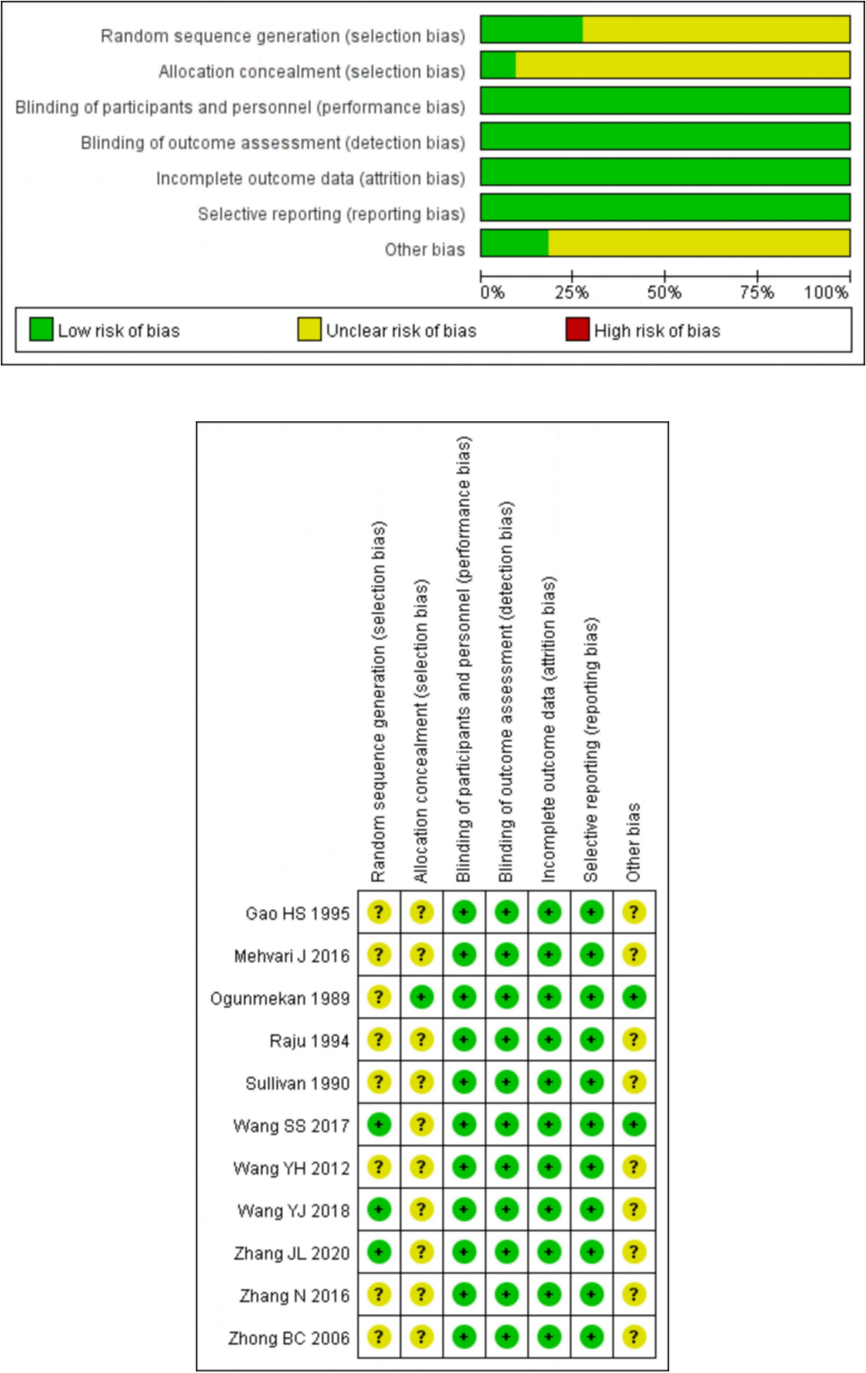
Risk of bias graph and summary.

For random sequence generation, 3 studies using random number tables were classified as low risk (21, 23, 24). The remaining 8 studies (13–15, 19, 20, 22, 25, 26), lacking details on randomization methods, had unknown bias risk. Regarding allocation concealment, 1 study using random coding by the research pharmacy department was low risk (13), while the rest, without clear details, were of unclear risk (14, 15, 19–26). Three studies employed double-blinding for implementers and participants (13–15). The other eight studies did not report on blinding; however, as the outcome measurement was judged unlikely to be affected, all were rated as low risk (19–26). None of the studies mentioned blinding of outcome assessors, yet as outcomes were unlikely to be impacted, all were classified as low risk for this aspect. All studies reported predetermined outcome measures with complete data, thus being evaluated as low risk for complete outcome data. In terms of reporting bias, 4 studies explained dropouts reasonably, rated as low risk (13–15, 19), and the others with no missing data were also low risk (20–26). Finally, due to insufficient data, the risk of other potential biases in all studies remained unknown. See Figure 2 for details.

### Efficacy

#### Reduction of seizure frequency

**Figure 3:**
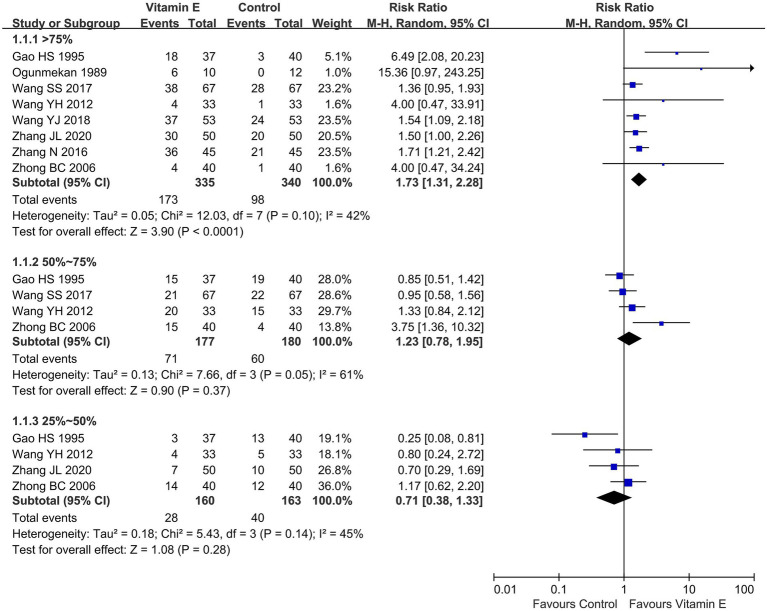
Reduction of seizure frequency between vitamin E and control groups.

Ten studies involving 759 patients reported the reduction of seizure frequency (13, 15, 19–26). One study demonstrated that following treatment, the seizure frequency in the vitamin E group decreased and was significantly lower than that in the control group (*p* < 0.01) (14). Eight studies reported a reduction of seizure frequency >75%, involving 675 patients (13, 20–26). With heterogeneity assessment in reducing seizure frequency by 50–75% showing significant variability across studies (*p* = 0.05, *I^2^* = 61%), a random-effects model was used for meta-analysis, in which the vitamin E group exhibited a significant advantage in reducing seizure frequency by over 75% compared to the control group (RR = 1.73, 95% CI (1.31, 2.28), *p* < 0.01. *I^2^* = 42%, heterogeneity *p* = 0.10). Four studies evaluated seizure frequency reductions of 50–75% (20–22, 26) and 25–50% (20, 22, 24, 26) separately. Meta-analysis revealed no statistically significant differences in reducing seizure frequency by 50–75% (RR = 1.23, 95% CI (0.78, 1.95), *p* = 0.37. *I^2^* = 61%, heterogeneity *p* = 0.05) or 25–50% (RR = 0.71, 95% CI (0.38, 1.33), *p* = 0.28. *I^2^* = 45%, heterogeneity *p* = 0.14) between the vitamin E and control groups (Figure 3). Because of overlapping data in studies reporting various degrees of epilepsy frequency decline, the total data were not pooled. In the 50–75% reduction subgroup, heterogeneity was 61%, potentially due to different treatment durations. Three studies had a treatment duration of 12 weeks (20–22), while the study with a 24 week treatment duration contributed most significantly to this heterogeneity (26).

**Figure 4:**
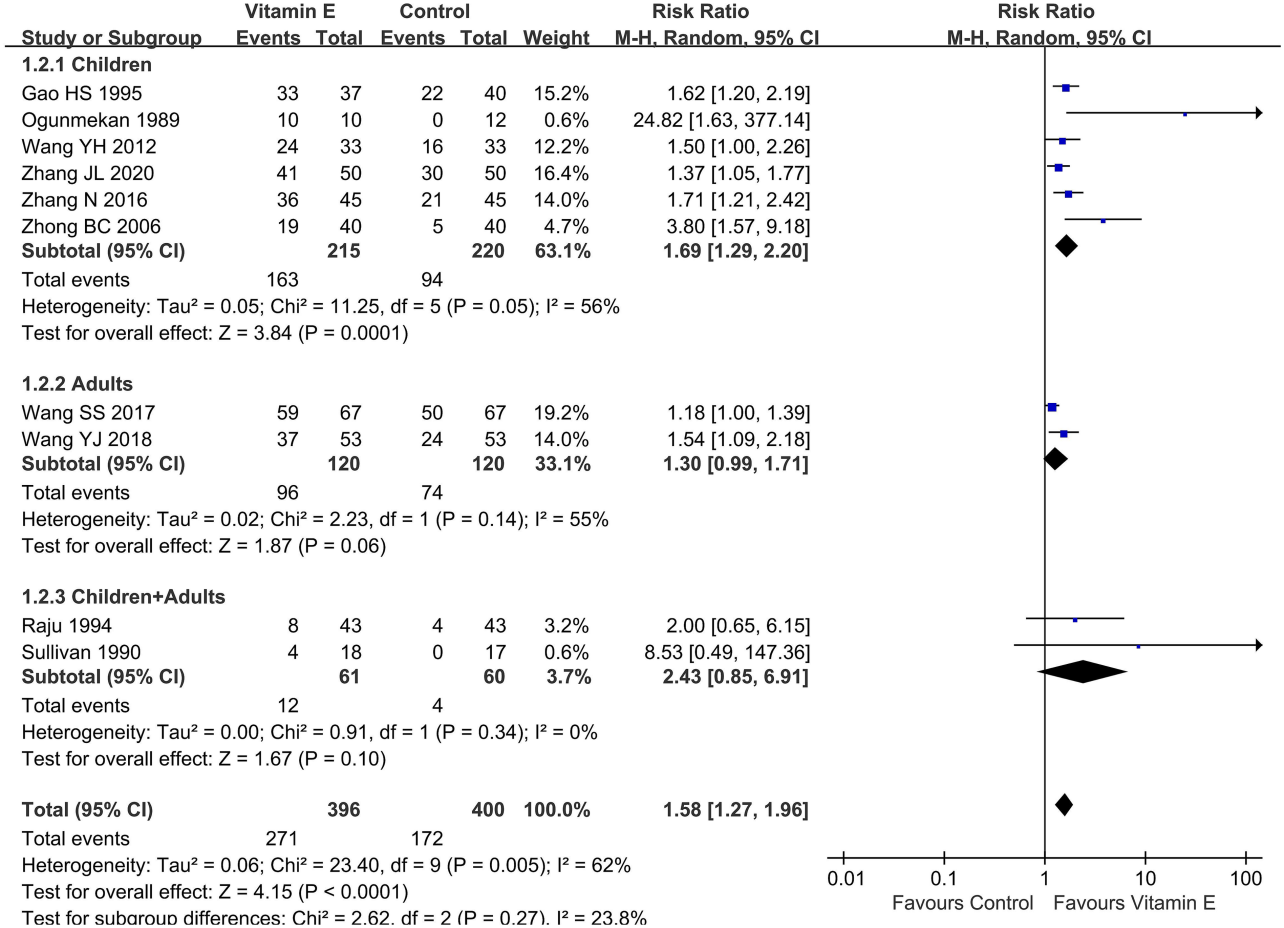
Subgroup analysis of reduction of seizure frequency>50% between vitamin E and control groups.

Given the varying definitions of epilepsy control in the included studies, with most using a > 50% frequency reduction as an efficacy indicator, we performed a subgroup analysis based on this metric. Ten studies (796 patients) (13, 15, 19–26) reported a > 50% seizure frequency reduction: six in children (435 patients) (13, 20, 22, 24–26), two in adults (240 patients) (21, 23), and two without differentiating between children and adults (121 patients) (15, 19). As the heterogeneity assessment indicated significant variability among the studies (*p* = 0.005, *I^2^* = 62%), a random-effects model was employed for meta-analysis. The meta-analysis unveiled a statistically significant difference in the achievement of a > 50% reduction in seizure frequency between the vitamin E group and the control group (RR = 1.58, 95% CI (1.27, 1.96), *p* < 0.01). In the subgroup analysis, the vitamin E group showed significant differences compared to the control group in children (RR = 1.69, 95% CI (1.29, 2.20), *p* < 0.01. *I^2^* = 56%, heterogeneity *p* = 0.05). However, in adults (RR = 1.30, 95% CI (0.99, 1.71), *p* = 0.06. *I^2^* = 55%, heterogeneity *p* = 0.14) or combining children and adults, no such significant trend was observed (RR = 2.43, 95% CI (0.85, 6.91), *p* = 0.10. *I^2^* = 0%, heterogeneity *p* = 0.34) (Figure 4).

### Changes in EEG

Six of the included studies reported on EEG improvements (13, 14, 20, 21, 23, 24). HSG (20) indicated that 52% of patients exhibited an improved EEG following vitamin E treatment. Ogunmekan (13) monitored the EEG of 7 patients before and after treatment. Among them, 4 patients showed enhanced background activity, 1 patient’s condition deteriorated from mild to moderate, and 2 patients showed no change. SSW (21) and YJW (23) reported that after treatment, the control group had significantly lower *θ* and *δ* waves, yet significantly higher *α* waves, compared to the vitamin E group. JLZ (24) demonstrated that the total effective rate of EEG in the vitamin E group was significantly higher than that in the control group. Mehvari et al. (14) reported a positive EEG decline rate of 50% in the vitamin E group (*n* = 32) and 12.1% in the control group (*n* = 33), with a significant difference between the two groups (*p* = 0.001). These results suggest that adjuvant vitamin E therapy is effective for epilepsy.

### The levels of T-Aoc and MDA in plasma

**Figure 5:**
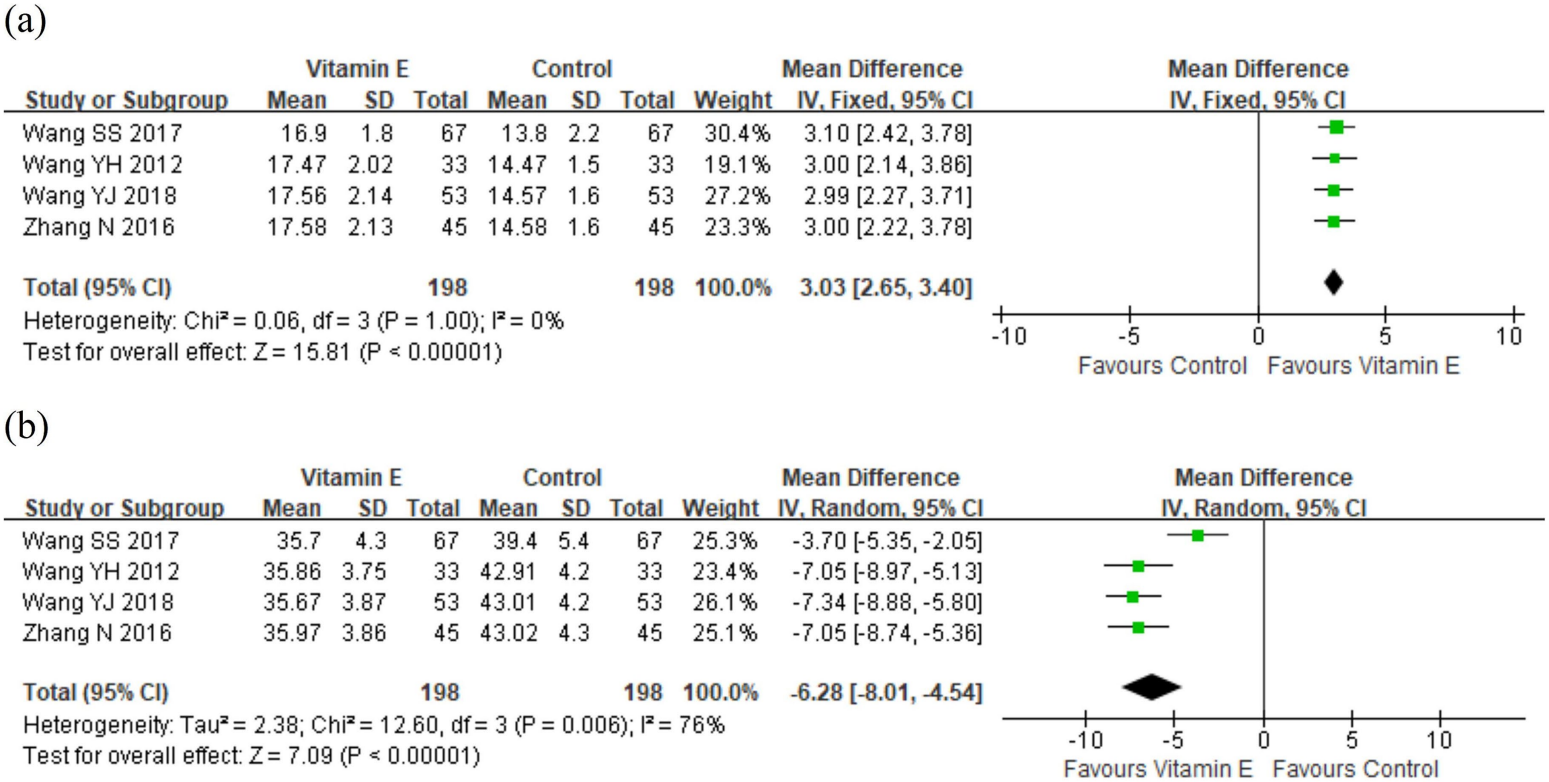
The levels of **(a)** T-Aoc and **(b)** MDA in plasma between vitamin E and control groups.

Four studies involving 396 patients reported plasma levels of T-Aoc and MDA to assess antioxidant capacity (21–23, 25). Meta-analysis revealed significant differences in T-Aoc (WMD = 3.03, 95% CI (2.65, 3.40), *p* < 0.01. *I^2^* = 0%, heterogeneity *p* = 1.00) and MDA (WMD = −6.28, 95% CI (−8.01, −4.54), *p* < 0.01; *I^2^* = 76%, heterogeneity *p* = 0.006) between the vitamin E and control groups (Figure 5). One study revealed that the mean increase in T-Aoc, catalase, and glutathione was significantly higher in the vitamin E group than in the control group (*p* < 0.05), while there was no significant difference in MDA between the two groups (14). These results indicated that vitamin E had stronger antioxidant capacity than the control group. The observed heterogeneity among the studies might be attributed to different detection methods: two studies used ferrous iron reduction (21, 23), while the other two employed colorimetry (22, 25).

### Safety

#### Total adverse events

**Figure 6:**
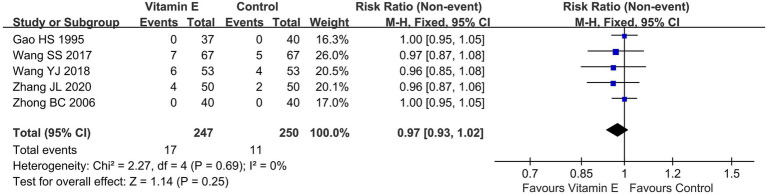
Total adverse events of included randomized controlled trials.

Of the 11 RCTs included in this analysis, merely three (21, 23, 24) reported on adverse events (AEs), two studies (20, 26) indicated that no adverse events occurred. The overall incidence rates of AEs in the vitamin E and control groups stood at 6.9 and 4.4%, respectively. A meta-analysis encompassing these five studies (20, 21, 23, 24, 26), which involved a total of 497 patients, demonstrated no statistically significant disparity in the overall AE rates between the vitamin E and control groups (RR = 0.97, 95% CI (0.93, 1.02), *p* = 0.25. *I^2^* = 0%, heterogeneity *p* = 0.69) (Figure 6).

#### Single adverse events

The risks of single AEs in the vitamin E group, in descending order, were nausea, dizziness, diarrhea, fatigue, and rash, while those in the control group were malaise, dizziness, sleepiness, diarrhea, and rash (Table 2).

**Table 2:** The details of single adverse events.

Adverse events	Wang et al. (21)		Wang (23)		Zhang et al. (24)		Total	
	Vitamin E (*n* = 67)	Control (*n* = 67)	Vitamin E (*n* = 53)	Control (*n* = 53)	Vitamin E (*n* = 50)	Control (*n* = 50)	Vitamin E (*n* = 170)	Control (*n* = 170)
Diarrhea	1	0	1	0	1	1	3(1.8%)	1(0.6%)
Dizzy	3	2	2	1	/	/	5(2.9%)	3(1.8%)
Fatigue	1	0	/	/	/	/	1(0.6%)	0(0%)
Malaise	0	2	0	2	/	/	0(0%)	4(2.4%)
Nausea	2	0	3	0	2	0	7(4.1%)	0(0%)
Rash	/	/	/	/	1	1	1(0.6%)	1(0.6%)
Sleepiness	0	1	0	1	/	/	0(0%)	2(1.2%)

### Assessment of the quality of the evidence

**Figure 7:**
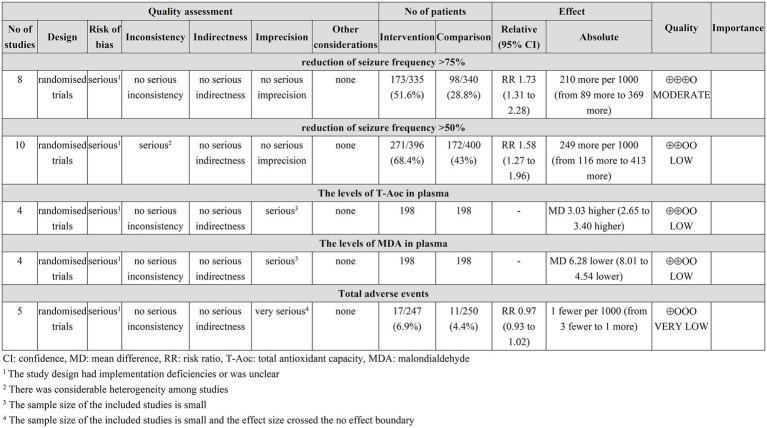
GRADEPro GDT quality assessment of outcomes.

The GRADEpro assessment results indicate that the reduction of seizure frequency >75% was moderate-quality evidence, the reduction of seizure frequency >50%, and the levels of T-Aoc and MDA in plasma were low-quality evidence, and the incidence of adverse events was very low-quality evidence, see Figure 7.

### Sensitivity analysis and publication bias

We performed a leave-one-out sensitivity analysis. Most outcome measurements remained stable, yet in the subgroup with a seizure frequency reduction < 50%, results varied. In the adult subgroup, with the exclusion of the SSW study (21), the vitamin E group showed a tendency towards an advantage compared to the control group (RR = 1.54, 95%CI (1.09, 2.18), *p* = 0.05). The data pointed to the erratic nature of the specified outcomes, making it essential to conduct further research.

This study employed the Nfs to assess the impact of publication bias on outcomes. The Nfs_0.05_ values for a 75 and 50% reduction in seizure frequency were 133.12 and 259.5, respectively. In the pediatric epilepsy subgroup, the Nfs_0.05_ for a 50% seizure frequency reduction was 129.44. The Nfs_0.05_ for the levels of T-Aoc and MDA in plasma were 359.36 and 310.56, respectively. As these Nfs_0.05_ values exceeded the number of included studies, the results were considered stable.

## Discussion

This study systematically evaluated the efficacy and safety of vitamin E in the treatment of epilepsy and provided a clinical basis for the clinical application of vitamin E as an adjuvant treatment for epilepsy. In the RCTs incorporated into this study, the majority of investigations delineated a reduction in seizure frequency >75% as a “significant clinical response” and a decrease >50% as a “clinical benefit.” Consequently, the results showed that the vitamin E group was superior to the control group in increasing the epilepsy control rate, especially in children. In adults, there was only a trend but no significant difference.

Oxidative stress is prevalent in epileptic patients, stemming from limited endogenous antioxidants and excessive free radical production. This imbalance leads to a disruption between pro-oxidant and antioxidant substances within the body. Notably, those with drug-resistant epilepsy experience more severe oxidative stress compared to those on one or two antiepileptic drugs (27). Prior research has shown elevated oxidative stress markers like MDA and decreased vitamin E levels along with reduced antioxidant status (measured by catalase, glutathione peroxidase, and T-Aoc, etc) in epileptic patients’ serum (28–31). Adjuvant vitamin E therapy can enhance blood T-Aoc levels and reduce MDA levels, potentially contributing to a higher epilepsy control rate. Thus, the plasma levels of T-Aoc and MDA serve as efficacy evaluation indicators for this treatment. In our study, the impact of vitamin E on these plasma levels in epileptic patients aligns with previous reports.

In safety assessment, meta-analysis indicated that there was no statistically significant difference in the rates of total AEs, suggesting that vitamin E was relatively safe epilepsy adjunct. However, a meta-analysis indicated that high-dose vitamin E supplementation (≥400 IU/d) was associated with an elevation in all-cause mortality (32). In addition, another study demonstrated that long-term consumption of 400 IU of vitamin E daily could lead to a 13% increase in the risk of heart failure and a 21% increase in the risk of hospitalization (33). Thus, careful dosage determination, especially for children, is crucial. Among the 11 RCTs, 2 used high vitamin E dosages (>400 IU) (15, 20). The GHS study (20) focused on children aged 5 to 14, and the Raju study (15) on patients aged 12 and over. In contrast, 6 included Chinese studies (21–26) used lower daily dosages: 100 mg for adults, 50 mg for children under 2, or 10 mg/kg for pediatric patients. This dosage variation may represent a critical factor affecting efficacy and safety, highlighting the need for further research on optimal dosing (such as age- and weight-standardized dosages) for different patient groups when using vitamin E in epilepsy treatment. Additionally, adverse events were reported in only 3 of the 11 studies, with an overall low incidence. This substantial limitation undermines the validity of safety conclusions. Future trials are urged to enhance adverse event reporting.

We comprehensively evaluated the efficacy and safety of vitamin E as an add-on therapy for epilepsy, analyzing its differential efficacy across populations and dosage variations among countries. Sensitivity and heterogeneity analyses were performed to offer a comprehensive view for clinical practice. Nevertheless, several limitations exist. Most included studies had an unclear bias risk: eight did not report the randomization method (13–15, 19, 20, 22, 25, 26), ten failed to explain allocation and concealment, and eight did not mention blinding (14, 15, 19–26). Flaws in random sequence generation may cause selection bias, potentially overestimating vitamin E’s efficacy if healthier patients are mistakenly assigned to the intervention group. Inadequate allocation concealment leads to performance bias, as group assignment knowledge among researchers/participants may skew outcome assessments (e.g., seizure frequency reporting). Lack of blinding heightens detection bias risk, especially for subjective outcomes like self-reported seizure improvement. Only five studies (14, 21–23, 25) reported serum T-Aoc and MDA levels, with three showing similar data (22, 23, 25). Moreover, three studies listed only one author (22, 23, 25). These factors potentially compromised the reliability of the results.

The subjects in the included RCTs of this study were mostly drug-refractory epilepsy patients (13–15, 19–21, 23, 26), mainly children (13, 20, 22, 24–26). The vitamin E treatment duration was typically 12 weeks (13, 20, 22, 24, 25). Considering safety, children over 2 years old were given 100 mg of vitamin E daily, and those under 2 years old received 100 mg every other day. These findings suggest that, for children with refractory epilepsy, vitamin E at this dosage and treatment duration as an adjunctive therapy might be beneficial. However, validation of this conclusion demands more high-quality research.

For the epileptic population, most current studies on vitamin E as adjunctive therapy for epilepsy have not distinguished between epilepsy types, precluding comparisons of vitamin E use differences between different epilepsy types (such as generalized vs. focal epilepsy). Additionally, since most studies on vitamin E as adjunctive therapy have been conducted in drug-resistant epileptic patients, differences in vitamin E efficacy between drug-resistant and drug-responsive epileptic patients cannot be compared. Future studies should explore these aspects to further identify the beneficiary populations of vitamin E adjunctive therapy for epilepsy.

## Conclusion

Vitamin E, when employed as an adjunctive therapy for epilepsy, especially drug-resistant epilepsy, effectively reduces seizure frequency, with a particularly notable impact in the pediatric population. The underlying mechanism is likely linked to its enhancement of antioxidant capacity. However, more research is required to validate its efficacy in adults. In terms of safety, vitamin E exhibits good safety characteristics and does not increase the incidence of AEs. Nonetheless, high-quality studies are still needed to firmly establish the role of vitamin E in antiepileptic treatment.

## Data Availability

The original contributions presented in the study are included in the article/Supplementary material, further inquiries can be directed to the corresponding author.
