# Supplementary material for: Efficacy and safety of vitamin E as adjunctive therapy for epilepsy: a systematic review and meta-analysis of randomized control trials
Source: Front Neurol. 2025 Jul 11;16:1628032. doi: 10.3389/fneur.2025.1628032 (PMC12289704; doi:10.3389/fneur.2025.1628032)
Supplement: Supplementary file 1 [file Table_1.DOCX]

**Table S1** Details for search strategy

| **Searches** | **Results** |
| --- | --- |
| **Pubmed** |  |
| ("Epilepsy"[Mesh] OR "Seizures"[Mesh] OR epilep*[Title/Abstract] OR seizure*[Title/Abstract] OR convuls*[Title/Abstract]) AND ("Vitamin E"[Mesh] OR "alpha-Tocopherol"[Mesh] OR vitamin E[Title/Abstract] OR tocopherol[Title/Abstract] OR alpha-tocopherol[Title/Abstract] OR"VE"[Title/Abstract]) | 345 |
| **Embase** |  |
| ('epilepsy'/exp OR 'seizure'/exp OR 'epilep*':ab,kw,ti OR 'seizure*':ab,kw,ti OR 'convuls*':ab,kw,ti) AND ('vitamin E'/exp OR 'vitamin E':ab,kw,ti OR 'tocopherol':ab,kw,ti OR 'alpha-tocopherol':ab,kw,ti OR 'VE':ab,kw,ti) AND 'human'/de | 1062 |
| **Cochrane** |  |
| #1. [mh Epilepsy] OR [mh Seizures]  #2. (epilep* OR seizure* OR convuls*):ti,ab,kw  #3. #1 OR #2  #4. [mh "Vitamin E"] OR [mh Tocopherols]  #5. ("vitamin E" OR tocopherol OR alpha-tocopherol OR VE):ti,ab,kw  #6. #4 OR #5  #7. #3 AND #6 | 205  **Trials115**  Cochrane Reviews83  Cochrane Protocols6  Clinical Answers1 |
| **CBM** |  |
| #1 "癫痫"[常用字段:智能] OR "癫痫发作"[常用字段:智能] OR "惊厥"[常用字段:智能]  #2 "维生素E"[常用字段:智能] OR "生育酚"[常用字段:智能] OR "维E"[常用字段:智能] OR "VE"[常用字段:智能]  #3 #1 AND #2  ( "癫痫"[常用字段:智能] OR "癫痫发作"[常用字段:智能]OR "惊厥"[常用字段:智能] AND "维生素E"[常用字段:智能] OR "生育酚"[常用字段:智能] OR "维E"[常用字段:智能] OR "VE"[常用字段:智能] | 590 |
| **CNKI** |  |
| SU=(癫痫 OR 癫痫发作 OR 惊厥) AND SU=(维生素E OR 维E OR 生育酚 OR VE) | 28 |
| **WanFang** |  |
| 主题:("癫痫"OR "癫痫发作" OR "惊厥" ) and 主题:("维生素E" OR "生育酚" OR "维E" OR "VE" ) | 201 |
| **VIP** |  |
| M=(针癫痫 OR 癫痫发作 OR 惊厥) AND M=(维生素E OR 维E OR 生育酚 OR VE) | 7 |
